# Chinese and Korean Characters Engage the Same Visual Word Form Area in Proficient Early Chinese-Korean Bilinguals

**DOI:** 10.1371/journal.pone.0022765

**Published:** 2011-07-27

**Authors:** Jian'e Bai, Jinfu Shi, Yi Jiang, Sheng He, Xuchu Weng

**Affiliations:** 1 Institute of Psychology, Chinese Academy of Sciences, Beijing, China; 2 Graduate University of Chinese Academy of Sciences, Beijing, China; 3 Department of Psychology, University of Minnesota, Minneapolis, Minnesota, United States of America; 4 School of Psychology, Southwest University, Chongqing, China; 5 Center for Cognition and Brain Disorders, Hangzhou Normal University, Hangzhou, Zhejiang, China; University of Leuven, Belgium

## Abstract

A number of recent studies consistently show an area, known as the visual word form area (VWFA), in the left fusiform gyrus that is selectively responsive for visual words in alphabetic scripts as well as in logographic scripts, such as Chinese characters. However, given the large difference between Chinese characters and alphabetic scripts in terms of their orthographic rules, it is not clear at a fine spatial scale, whether Chinese characters engage the same VWFA in the occipito-temporal cortex as alphabetic scripts. We specifically compared Chinese with Korean script, with Korean script serving as a good example of alphabetic writing system, but matched to Chinese in the overall square shape. Sixteen proficient early Chinese-Korean bilinguals took part in the fMRI experiment. Four types of stimuli (Chinese characters, Korean characters, line drawings and unfamiliar Chinese faces) were presented in a block-design paradigm. By contrasting characters (Chinese or Korean) to faces, presumed VWFAs could be identified for both Chinese and Korean characters in the left occipito-temporal sulcus in each subject. The location of peak response point in these two VWFAs were essentially the same. Further analysis revealed a substantial overlap between the VWFA identified for Chinese and that for Korean. At the group level, there was no significant difference in amplitude of response to Chinese and Korean characters. Spatial patterns of response to Chinese and Korean are similar. In addition to confirming that there is an area in the left occipito-temporal cortex that selectively responds to scripts in both Korean and Chinese in early Chinese-Korean bilinguals, our results show that these two scripts engage essentially the same VWFA, even at the level of fine spatial patterns of activation across voxels. These results suggest that similar populations of neurons are engaged in processing the different scripts within the same VWFA in early bilinguals.

## Introduction

Reading is an important skill in modern society. There is evidence for an abstract representation of visual word form. Recent brain imaging studies have identified a region in the ventral occipito-temporal cortex that is consistently activated by words in normal readers, regardless of the position, size, color, font, or case used [1], [2], [3], [4], [5], [6], [7] and neuropsychological studies show that patients with lesions in this area are selectively impaired in reading letters and words [8], [9], [10], [11], [12], [13], [14]. Cohen and his colleagues labeled this area in the left fusiform gyrus (Talairach coordinate: −43,−54,−12) the Visual Word Form Area (VWFA) and suggested that it is specialized in processing the abstract visual form of words [2], [3].

VWFAs have been identified for processing visual words in both alphabetic writing system, such as English, Hebrew, French [4], [15], [16] and non-alphabetic writing system, like Chinese [17], [18], [19]. There is some indication that different alphabetic writing systems could engage the same VWFA region in occipito-temporal cortex. For example, in the research of Baker et al., they found that in Hebrew-English bilinguals, both Hebrew and English activated an area in the occipito-temporal cortex and there was considerable overlap between these two regions [15]. However, even though visual words from both alphabetic and non-alphabetic writing systems seem to be processed in the lateral fusiform region, at a fine spatial scale whether a non-alphabetic writing system, such as Chinese, engages the same VWFA as alphabetic writing systems is still unclear.

Written Chinese is thought to be a unique script. Compared to visual words in alphabetic scripts, the logographic Chinese character not only has different surface form, but also has dramatically different orthography properties, including orthographic rules and orthographic transparency. Chinese characters have a square shape and consist of radicals that are combinations of strokes. One or more radicals combined together form a Chinese character. More importantly, unlike alphabetic scripts, Chinese characters do not map graphemes to phonemes but map a whole character to a spoken syllable which is usually a meaningful morpheme. The mapping from graphic unit to spoken syllable is arbitrary. These unique properties of Chinese script make it require more complicated orthographic processing than even the deepest alphabetic orthography in alphabetic scripts [17], [20] and different from alphabetic scripts in phonological and semantic representations in the brain [21], [22], [23], [24], [25], [26], [27], [28], [29]. Therefore, it is important to answer whether the different mapping relationships from word form to phonological and semantic information for Chinese characters compared with alphabetic scripts impose different demands on the visual word form processing and as a result leading to different VWFAs.

Such a question has been addressed at the gross level. A number of studies have shown that the VWFA for Chinese characters in the occipito-temporal cortex is roughly consistent with the area identified for alphabetic scripts [17], [19], [30]. And in a meta-analysis study, Bolger and colleagues compared the location of the VWFA for Chinese characters and alphabetic writing systems. They examined 25 studies in English and other Western European languages that use an alphabetic writing system and 9 studies of native Chinese reading and found that there was about a 6 mm peak distance in Talairach space between Chinese and alphabetic writing systems in the left fusiform gyrus (Chinese characters: −49, −53, −10; Western words: −46, −56, −15). Therefore, they considered that the VWFA across writing systems had consistent localization [20]. In the study of Chee and colleagues investigating whether cortical organization for a first language and second language is different in English-Chinese bilinguals, they found some degree of overlapped activation in the basal temporal area (BA37) for Chinese and English at the single word level [25].

However, although these studies provide support that for different writing systems, the VWFA will fall in the same general area in the brain, they did not answer the question of whether in the same brain, the same VWFA is engaged for very different writing systems. Considering the limitation of meta analysis method and the very small size of VWFA [15], although the meta analysis result showed that VWFA for Chinese character in the occipito-temporal cortex was near the VWFA for alphabetic scripts, it is limited to confirm whether VWFA for Chinese character located in the same or adjacent area as the VWFA for alphabetic writing systems. Moreover, these studies only compared the location and response intensity of VWFAs for Chinese characters and alphabetic scripts and lacked the spatial detail with regard to the VWFA to answer the questions of whether the same or different VWFA was engaged for the two types of written words.

Therefore, we carried out our experiment to investigate this question in detail. We believe it is critical to investigate this question using a within-subject design, i.e., in bilinguals. We compared activations to Chinese vs. Korean characters in Chinese-Korean bilinguals. The advantage of comparing Chinese vs. Korean is that although Korean syllable characters have a square visual shape similar to Chinese character, as an alphabetic script, Korean is very different from Chinese in orthography. Korean consonant and vowel letters are phonemic letters, most of which can find their equivalents in English. There is a very regular and transparent correspondence between print and sound in Korean characters. Therefore, Korean characters have very shallow orthography depths and very regular orthography rules [31]. In addition, we controlled the age of acquisition of Chinese and Korean to avoid potential differences induced by the effect of the age of acquisition of their first and second languages [25], [32] and proficiency of these two languages for all participants. Moreover, in addition to the comparison of location, response intensity and voxel numer of VWFAs for Chinese and Korean characters, a multi-voxel pattern analysis (MVPA) was also used to further investigate spatial pattern of activity in the VWFA region for Chinese characters and Korean characters, which could provide rich information about the representation of these two writing systems. With these multiple approaches, we aimed to obtain a clear picture of whether Chinese characters and Korean alphabetic scripts were processed by the same VWFA in the left fusiform gyrus.

## Materials and Methods

### Ethics statement

The experimental procedure was approved by the IRB of the Institute of Psychology, Chinese Academy of Sciences. All participants provided written, informed consent before taking part in the fMRI experiment.

### Participants

A total of sixteen healthy proficient early Chinese -Korean bilingual volunteers took part in the fMRI experiment. Data from two subjects were excluded from analysis for falling asleep during the scan. The mean age of the 14 remaining subjects was 22 years (range = 19–24; 13 females and 1 male). All subjects were Chinese whose first language was Korean. All subjects acquired the spoken language of their first language (Korean) and second language (Chinese) before 5 years of age and learned to read in these two scripts before 9 years of age when they were in elementary school. We examined the proficiency of these two languages in each subject. When tested, all subjects were able to show mastery on more than 3000 Chinese characters which are necessary for normal reading. They were able to speak in Chinese and read aloud an article from Chinese newspaper, proficiently. All participants were undergraduate students in Beijing. Chinese was the language used in most courses and examinations (seven of the fourteen participants majored in Korean and they had half of their courses taught and examined in Korean). Chinese was the official language and most students in their universities spoke Chinese. Both Chinese and Korean were used in their normal life. Therefore, all the participants in our study were proficient early bilinguals.

All participants were fully right-handed and had normal or corrected-to-normal vision. None had neurological or psychiatric history.

### Stimuli and Procedure

There were four categories of stimuli used in our study: single Chinese characters, single Korean characters, unfamiliar Chinese faces and line drawings of common objects. Written characters were compared with faces and line drawings to reveal category selectivity for visual words in the VWFA [6], [12], [33], [34]. Face and line drawings are thought to have a similar level of visual complexity with words. In particular, faces have the best category selectivity in the fusiform area known as the Fusiform face area (FFA) [35] and can serve as a good contrast stimuli.

There were 60 images for each of the four categories of stimuli. Chinese characters used had high frequencies (>300 per million) and each had 6–8 strokes; each Korean character was chosen from primary Korean vocabulary and also had 6–8 strokes; line drawings of objects included buildings, tools and furniture; images of faces were black-and-white and half were male. The visual angle of each image was about 1.8 degrees in width and 2.4 degrees in height.

**Figure 1:**
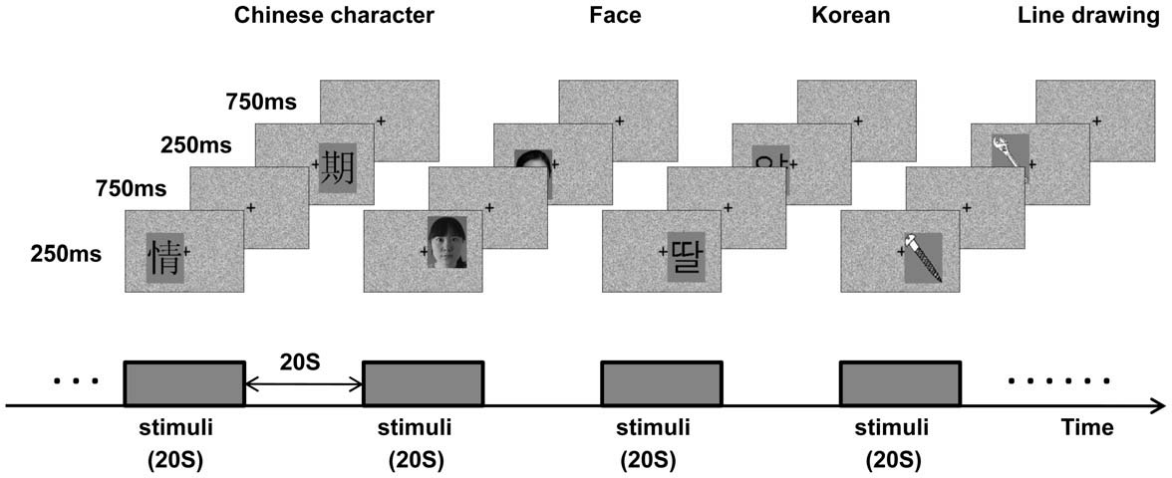
Schematic depiction of the experimental paradigm. In block-design runs, subjects viewed Chinese characters, Korean characters, unfamiliar faces and line drawings in separate blocks. In each block 20 stimuli were presented and each stimulus was presented for 250 ms with an inter-stimulus interval of 750 ms. Each run contained 12 blocks with 3 blocks for each category of stimuli.

The stimuli were presented in separate blocks, in a block-design paradigm with a simple content-irrelevant position judgment task. Each block lasted 20 s and consisted of 20 stimuli of the same category. Each stimulus was presented for 250 ms with an inter-stimulus interval (ITI) of 750 ms. A central fixation point in a rectangular area with a visual angle of 6.4 degrees in width and 4.6 degrees in height was present throughout each run. Participants were required to fix their eyes on the center point throughout each run. All stimuli pictures appeared pseudo-randomly in the rectangular area within each block. The center of each stimulus was slightly shifted from the center fixation point and participants were asked to judge whether the center of the picture was to the right or the left of the fixation point and respond with left or right key. Each run contained 12 blocks with 3 blocks for each category of stimuli. There was a 20 s interval between blocks and in the beginning and end of the run when participants only fixated on the fixation point and no task was required. Each run lasted 500 s. Each participant was scanned for 4 runs (Figure 1).

### MRI data acquisition

Brain images were obtained on a 3T Siemens Trio scanner with an eight-channel phase-array coil at Beijing MRI Center for Brain Research. The stimuli were generated on a PC and projected via a LCD projector onto a tangent screen located in the scanner. Subjects viewed the stimuli through a mirror located above their eyes. Functional images were acquired with an EPI sequence with parameters as follow: 13 slices approximately parallel to the base of the temporal lobe and covering the occipital lobe,occipito-temporal area and most of the temporal lobe; 4.0 mm slice thickness with no gap; field of view, FOV = 220×220 mm^2^; Matrix = 64×64; repetition time, TR = 2000 ms; echo time, TE = 30 ms; flip angle, FLA = 90°. For each subject, a high-resolution 3D structural data set (3D MPRAGE; 1×1×1.33 mm^3^ resolution, 144 slices and 1.33 mm slice thickness with no gap) was acquired for localization and visualization of the functional data in the same session after the functional runs. Parameters were as follows: FOV = 240×240 mm^2^, Matrix = 256×256, TR = 2530 ms, TE = 3.37 ms, IT = 1100 ms, FLA = 12°.

### MRI data analysis

Data were analyzed with BrainVoyager QX (Brain Innovation) software. Functional volumes in all runs for each participant were preprocessed, including slice scan timing correction, 3D motion correction and temporal filtering (High pass filter 0.006 Hz). All participants' head motion within any fMRI run was less than 2 mm. The functional data were then aligned with the anatomical data and transformed into the Talairach space. The voxel size of functional data was resampled to 1×1×1 mm^3^.

The change of BOLD signals induced by Chinese characters and Korean characters were calculated against the fixation blocks for each participant.

Activation maps induced by Chinese characters and Korean characters were generated using a GLM (general linear model) procedure at the individual level in a common Talairach space. Partly because of the mixed varieties of objects used in the line drawing stimuli, contrasting the characters with line drawing stimuli yielded less consistent activation map in the fusiform region. Thus we compared the activation of characters to that of the faces. The area that responded more strongly to the Chinese characters than faces in left occipito-temporal area was defined as VCFA (Visual Chinese character Form Area) and the area that responded more strongly to the Korean characters than faces was defined as VKFA (Visual Korean character Form Area). Further quantitative analysis was carried out in these two areas (VCFA and VKFA).

We used individual analyses to conduct a within-subject comparison of these two scripts to avoid potential confounding factors in comparison of different subject groups. We compared the activation peak point distance, the overlap and the voxel numbers between VCFA and VKFA and response amplitude of Chinese and Korean characters in these two regions.

Moreover, we also performed a multi-voxel pattern analysis (MVPA) of the data, to examine the detailed spatial pattern of activity across voxels [36]. We used the data of the first and third runs to define the ROI and data from the second and the fourth runs for correlation based MVPA. First, in each subject, with a GLM based procedure, we contrasted the Chinese characters and Korean characters together with fixation. We took the point with the highest statistical value (t value) as a center to draw a sphere with 6 mm radius. All the voxels within this sphere were included for MVPA analysis. Then, using the correlation based multi-voxel pattern analysis (MVPA) [37], we calculated the correlation coefficients between the pattern of response evoked by each category during the second run and the pattern of response evoked by each category during the fourth run. Our main interest is to investigate how similar the pattern of activation was between Chinese and Korean characters (C-K). In order to make this evaluation, we also obtained four types of within-category correlation coefficients: (Chinese-Chinese, Korean-Korean, Linedrawing-Linedrawing and Face-Face) and five between-category correlation coefficients (Chinese-Linedrawing; Korean-Linedrawing, Chinese-Face, Korean-Face, and Face-Linedrawing). Thus the correlation coefficient C-K could be evaluated against a set of within-category coefficients and a set of between-category coefficients.

## Results

In each subject, we first identified regions selectively activated by Chinese characters and Korean characters in contrast to faces in the left occipito-temporal cortex, respectively. In each participant we could find a selective cortical area for Chinese characters and one for Korean characters in the left occipito-temporal sulcus. We defined the selective area for Chinese characters as VCFA and the selective area for Korean characters as VKFA, and found that VCFA and VKFA had a high degree of overlap.

**Figure 2:**
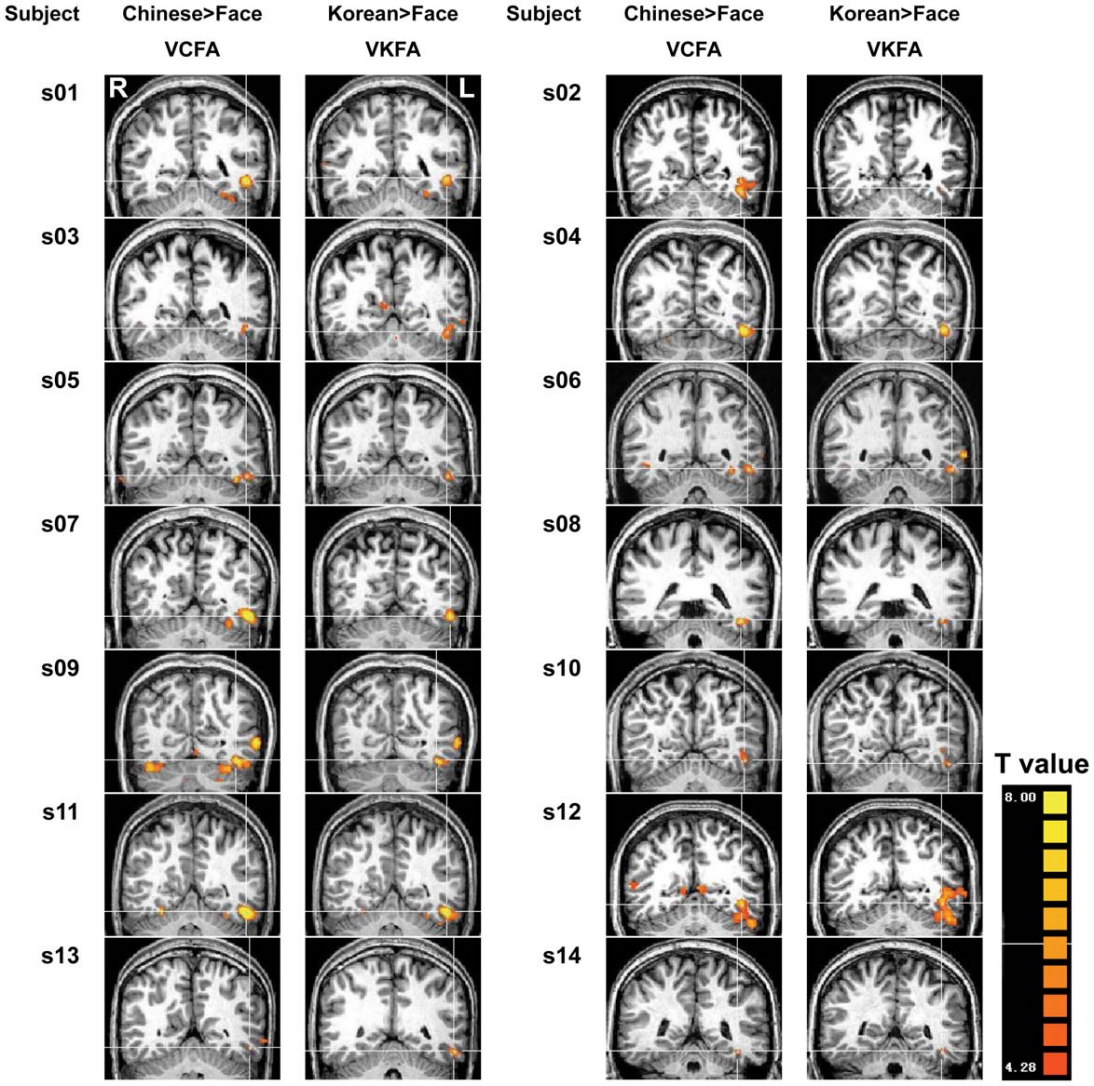
Selectively activated area for Chinese characters and Korean characters in the left occipito-temporal cortex. Brain activation maps in Talairach space of each subject show the activation of Chinese characters and Korean characters when contrasted with faces, respectively (q(FDR)<10^−3^). The right side of the image corresponds to the left hemisphere. The cross in each image is centered at the coordinate for the peak category selective response point. The color bar shows the t values.

Figure 2 shows the activation maps for all subjects which graphically conveys the degree of consistency of VCFA and VKFA across these two writing systems. Using the same threshold (q(FDR)<10^−3^), there were 680 voxels in VCFA and 425 voxels in VKFA when averaged across subjects. Paired sample t-test showed there was no significant difference between VCFA and VKFA in the number of voxels (*t*(13) = 2.077, *p* = 0.058). The levels of response to Chinese characters and to Korean characters in VCFA and VKFA respectively were also similar. Paired sample t-test showed that the beta value of the responses for Chinese characters and Korean characters had no significant differences (*t*(13) = −0.387, *p* = 0.705 in VCFA and *t*(13) = −0.271, *p* = 0.791 in VKFA).

In the following sections, we describe further quantitative analysis of the spatial relationship between VCFA and VKFA.

### Peak category selective response point for Chinese characters and peak category selective response point for Korean characters

In identifying the VCFA and VKFA, we contrasted response to Chinese and Korean characters with that to faces. The point with the highest statistical value (t value) in the contrast between Chinese characters / Korean characters and faces was defined as the peak category selective response point. Then, we extracted peak category selective point coordinates in VCFA and VKFA, respectively. All participants showed that the peak category selective response point in VCFA and the peak category selective response point in VKFA were located in almost the same coordinates in individual analysis. Table 1 shows the peak coordinates for Chinese characters and Korean characters in VCFA and VKFA, respectively.

**Table 1:** Coordinate of VCFA and VKFA and the peak point distance (mm) between these two areas.

Subject	Peak of VCFA	Peak of VKFA	X-axis distance	Y-axis distance	Z-axis distance	Peak distance
s05	(−48,−52,−11)	(−48,−52,−11)	0	0	0	0
s08	(−42,−40,−11)	(−42,−40,−11)	0	0	0	0
s09	(−39,−64,−8)	(−39,−64,−8)	0	0	0	0
s11	(−48,−49,−15)	(−48,−49,−15)	0	0	0	0
s12	(−42,−52,−8)	(−42,−52,−8)	0	0	0	0
s04	(−45,−60,−8)	(−45,−58,−8)	0	2	0	2
s01	(−48,−49,−5)	(−48,−49,−2)	0	0	3	3
s06	(−48,−49,−5)	(−51,−49,−5)	−3	0	0	3
s07	(−51,−55,−8)	(−51,−58,−8)	0	−3	0	3
s02	(−42,−55,−14)	(−42,−52,−11)	0	3	3	4.2
s03	(−48,−49,−8)	(−48,−52,−11)	0	−3	−3	4.2
s10	(−45,−55,−8)	(−48,−55,−11)	−3	0	−3	4.2
s14	(−39,−43,−11)	(−42,−46,−11)	−3	−3	0	4.2
s13	(−51,−49,−8)	(−54,−43,−11)	−3	6	−3	7.4
Average	(−45.4,−51.5,−9.1)	(−46.3,−51.4,−9.4)	−0.9	0.1	−0.3	1.0

We further computed the distance between the peak category selective response point in VCFA and the peak category selective response point in VKFA in each subject. The results also confirm that these two peaks are very close in each subject. Indeed, in five out of the fourteen subjects these two points (peak VCFA and peak VWFA) are in the identical location (Table 1). Across subjects, the average peak category selective response point in VCFA is (−45.4, −51.5, −9.1) and in VKFA is (−46.3, −51.4, −9.4) in Talairach space. The average distance of the two peak category selective response points is only about 1 mm (average distance in x, y, z is 0.9 mm, 0.1 mm, and 0.3 mm respectively).

As noted in the methods section, contrasting the characters with line drawing stimuli yielded less consistent activation map in the fusiform region. Only 5 of the 14 subjects showed significantly higher responses to characters than line drawing objects in the fusiform region. We believe it is partly because of the rich varieties of objects used in the line drawing stimuli. To test the robustness of the peak location results, we also obtained the peak response to Chinese characters and to Korean characters using fixation as the baseline. In 9 of the 14 subjects, the locations of peak responses (with the maximal t value) to Chinese and Korean characters are the same. Across subjects, the average peak location in Talairach space for Chinese characters is (−41.8, −55.4, −8.9) and the average peak point for Korean characters is (−41.6,−56.1,−9.1). Compared to the peak points identified with characters vs. faces, the peak point for characters identified with fixation as baseline shifted about 4 mm medially, but the distance between this two peak point remains very small, only about 0.8 mm.

### Overlap index

In addition to obtain the selective peaks for VCFA and VKFA which coincide with each other, we also calculated the degree of overlap between these two selective areas in the left occipito-temporal cortex, using the following formulae.

- ROIa ∩ ROIb / ROIa
- ROIa ∩ ROIb / ROIb
- ROIa ∩ ROIb / ROIa∪ROIb

For formulae A and B, the number of voxels for a single category is used as the denominator. For formulae C, the number of voxels for ROIa OR ROIb is used as the denominator [38]. In our study, the number of active voxels in VCFA (using a threshold q(FDR)<10^−3^) was used as ROIa, alternatively, the number of active voxels in VKFA (using a threshold q(FDR)<10^−3^) was used as the denominator ROIb. If there were 100 voxels in VCFA and 200 voxels in VKFA, and the number of overlapping voxels for VCFA AND VKFA was 50 voxels, then an overlap index in the case of formula A is 50%, an overlap index in the case of formula B is 25%, and overlap index in the case of formulae C is 20%.

Table 2 shows the overlap index results calculated using formula A, B and C, for both individuals and the group average. In most of the 14 subjects, the overlap of VCFA and VKFA is considerable. Further group analysis shows that the overlap index of these two areas is 79.06% when VKFA is used as the denominator. This means that almost 80% of voxels in VKFA can also be found in VCFA. When VCFA is used as the denominator, the overlap index between VCFA and VKFA is 49.41%. Even when VCFA OR VKFA is used as the denominator, the overlap index is still more than 40%. These results indicate that there is considerable overlap between these two areas.

**Table 2:** Voxel number of VCFA, VKFA, VCFA ∩ VKFA and VCFA ∪ VKFA and overlap indexes between VCFA and VKFA.

Subject	VCFA	VKFA	VCFA∩VKFA	VCFA∪VKFA	Overlap index1	Overlap index2	Overlap index3
s01	977	626	615	988	62.95%	98.24%	62.25%
s02	762	39	39	762	5.12%	100.00%	5.12%
s03	140	381	84	437	60.00%	22.05%	19.22%
s04	1816	817	806	1827	44.38%	98.65%	44.11%
s05	248	218	196	270	79.03%	89.91%	72.59%
s06	281	160	144	297	51.25%	90.00%	48.48%
s07	2029	828	826	2031	40.71%	99.76%	40.67%
s08	449	87	87	449	19.38%	100.00%	19.38%
s09	677	313	313	677	46.23%	100.00%	46.23%
s10	100	129	18	211	18.00%	13.95%	8.53%
s11	1181	900	852	1229	72.14%	94.67%	69.32%
s12	705	886	667	924	94.61%	75.28%	72.19%
s13	91	452	15	528	16.48%	3.32%	2.84%
s14	67	110	43	134	64.18%	39.09%	32.09%
Average	680	425	336	769	49.41%	79.06%	43.69%

Note: Overlap index1, VCFA ∩ VKFA / VCFA; overlap index2, VCFA ∩ VKFA / VKFA; overlap index3, VCFA ∩ VKFA / VCFA ∪ VKFA.

### Spatial patterns of response to Chinese and Korean characters

The results described above show that the same region in the left occipito-temporal cortex is engaged in processing Chinese characters and Korean characters. However, the VCFA and VKFA could occupy the same spatial location but may still have different spatial sub-regions responsible for processing the two scripts. Since the fine scale spatial pattern of activation within a region could provide a more sensitive measure of a region's response property, we conducted Multi-voxel-pattern analysis (MVPA) to further examine the detailed activation pattern in response to these two types of scripts.

MVPA was performed in the independently defined ROI (data from odd runs were used to define the ROI and data from even runs were used to compute the correlation based MVPA. See the Methods section for details). The ROI was a spherical volume in the left mid-fusiform cortex, with 6 mm radius and centered at the peak point of (Chinese+Korean) vs. fixation contrast. For each participant, we extracted the BOLD signal in each voxel for Chinese and Korean characters by averaging time points 8–12 s after the onset of each character block.

In addition to the correlation coefficient between Chinese character and Korean characters, we also calculated four types of within-category correlation coefficients (Chinese-Chinese, Korean-Korean, Linedrawing-Linedrawing, and Face-Face) and five types of between-category correlation coefficients (Chinese-Linedrawing, Korean-Linedrawing, Chinese-Face, Korean-Face, and Face-Linedrawing).

**Figure 3:**
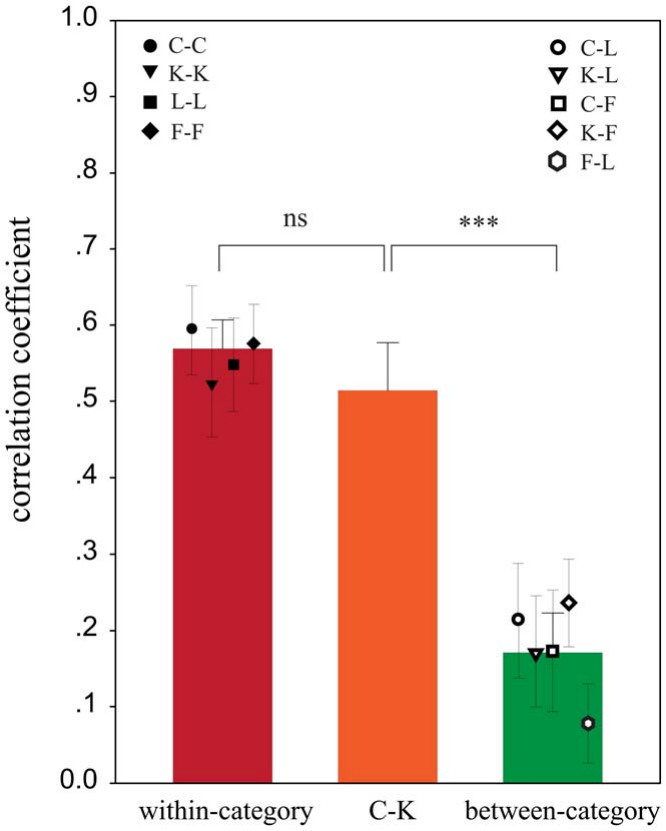
Correlation coefficients for within-category stimuli (left bar, C-C, K-K, L-L, F-F) and correlation coefficients for between-category stimuli (right bar, C-L, K-L, C-F, K-F, F-L). The correlation coefficient C-K (middle bar) is not significantly different from the within-category correlations, but significantly different from the between category correlations (*p* = 0.000).

Results show that the four within-category correlation coefficients (C-C = 0.60, K-K = 0.53, L-L = 0.56 and F-F = 0.58) are substantially higher than the five between-category correlation coefficients (C-L = 0.21, K-L = 0.17, C-F = 0.17, K-F = 0.24, F-L = 0.07). The correlation coefficient of C-K is 0.51. To evaluate whether Chinese characters and Korean characters were treated as one category or two different categories, we compared correlation coefficient C-K with with-category correlation coefficients and between-category correlation coefficients. Paired-sample t test analysis indicated that there was no significant difference between correlation coefficient C-K and the mean within-category correlation coefficient (*t*(13) = 1.482, *p* = 0.162) but the correlation coefficient C-K was significantly different from the mean between-category correlation coefficient (*t*(13) = 6.125, *p* = 0.000) (Figure 3). Thus, at least based on the pattern of activation across voxels in the ROI, Chinese and Korean characters were apparently treated as belonging to the same category.

## Discussion

The purpose of this study is to investigate whether different writing systems engage the same VWFA in the left occipito-temporal cortex, in particular contrasting between a logographic script (Chinese) and an alphabetic writing system (Korean). By selecting proficient early Chinese-Korean bilinguals to participate in our study, we conducted a within subject experiment and adopted a multi-analysis approach. We found that in each participant, when compared with faces, both Chinese characters and Korean characters activated an occipito-temporal area in the coordinate location reported for VWFA in studies using alphabetic scripts [2], [3], [15], [34], which is consistent with the result of a meta-analysis study [20] and analyses at gross scales [17], [19], [30]. More importantly, although there is dramatic differences in orthography between Chinese characters and Korean characters, we found that the two types of scripts essentially engaged the same VWFA. Specifically, at both the individual and group levels, we observed similar locations and response amplitudes between the VCFA and VKFA, and their extents largely overlapped. These results substantiate the view that VWFA for Chinese characters is located in the same region as alphabetic writing systems [17], [20]. In addition, the MVPA revealed that the spatial patterns of activation for these two character types were as similar as patterns generated from the same stimulus category, which suggests that even at a spatial scale of individual voxels, VCFA and VKFA are not differentiated.

There are two primary views about what information of visual words is represented in the VWFA. One view is that the VWFA primarily represents perceptual information (i.e., the form) of visual words (the perceptual hypothesis) [14], while the other is that it mainly serves as an interface between the visual form and high level phonologic and semantic representation (the interface hypothesis) [39]. There is ample evidence that the neural basis underlying Chinese logograph reading, and the phonologic and semantic representation of Chinese characters in the brain are different from that of alphabetic scripts [21], [22], [23], [24], [25], [26], [27], [28], [29]. If the VWFA mainly serves as the interface of the visual and linguistic systems, it is possible for individuals who can read both Chinese and an alphabetic script, the two types of visual word forms may be processed by different VWFAs. However, our results indicated that the coordinates and spatial pattern of activation of this specialized visual word area in left occipito-temporal cortex for Chinese characters and Korean characters were almost the same, despite their different high level phonology and semantic representations. Thus our results do not support the interface hypothesis. Instead, our findings favor the perceptual hypothesis, which suggest that the selectivity of VWFA for words is dependent on the degree of expertise but not the type of scripts. Individuals who have the same degree of expertise for two scripts, such as proficient early bilinguals in our study, use the same VWFA to process logographic and alphabetic scripts, although they largely differ in the mapping between visual and sound forms. The important role of the age of reading acquisition or proficiency may also explain why some studies found different activations to different scripts. For example, in a study of native Chinese who learned to read English as the second language much later, Chinese and English showed different activation in the extrastriate cortex [40]. Similarly, Nakamura and colleagues reported a difference in the VWFA for Japanese Kana (a regular syllable script) and Kanji (a logographic script) in native Japanese [41], as it has been shown that Kana is more frequently used than Kanji [42]. However, it has also been suggested that compared with Kana, the more holistic processing of Kanji may have contributed to the observed differences [43].

Orthography is thought to affect early stages of visual word processing [44], [45], [46]. The VWFA, according to some researchers, may be sensitive to orthographic regularity [3], [14]. It would not be unreasonable to expect that the unique orthographic properties of Chinese characters require different computational mechanisms compared to alphabetic writing systems. The fact that we found no difference in the VWFA for processing Chinese and Korean characters suggest that additional brain areas are involved in processing orthographic information. For example, the left middle frontal gyrus was consistently found to be involved in reading Chinese [17], [19], [21], [22], [23], [27], [47], a region likely recruited to process the complex visuo-spatial configurations with the Chinese orthography.

In summary, our results demonstrate that a common VWFA is engaged in processing the logographic Chinese and the alphabetic Korean characters, two orthographically distinct scripts. The spatial pattern of activation across voxels in VWFA induced by these two scripts were also similar, suggesting that at the spatial scale of voxels, the different written systems in early bilinguals engage similar neuronal populations in processing their visual forms.
